# The Older Persons’ Transitions in Care (OPTIC) study: pilot testing of the transition tracking tool

**DOI:** 10.1186/1472-6963-13-515

**Published:** 2013-12-14

**Authors:** Robert Colin Reid, Garnet E Cummings, Sarah L Cooper, Stephanie L Abel, Laura J Bissell, Carole A Estabrooks, Brian H Rowe, Adrian Wagg, Peter G Norton, Mike Ertel, Greta G Cummings

**Affiliations:** 1 School of Health and Exercise Sciences, University of British Columbia’s Okanagan campus, 3333 University Way, ART, Kelowna, British Columbia V1V 1V7, Canada; 2 Department of Emergency Medicine, Faculty of Medicine and Dentistry and School of Public Health, University of Alberta, Edmonton, Alberta, Canada; 3 Faculty of Nursing, University of Alberta, 5-110 Edmonton Clinical Health Academy, 11405 87 Avenue, Edmonton, Alberta T6G 1C9, Canada; 4 Division of Geriatric Medicine, Department of Medicine, Faculty of Medicine and Dentistry, University of Alberta, Edmonton, Alberta, Canada; 5 Department of Family Medicine, Faculty of Medicine, University of Calgary, Calgary, Alberta, Canada; 6 Kelowna General Hospital, Interior Health Authority, Kelowna, British Columbia, Canada

**Keywords:** Transfers, Transitional care, Transition tracking, Nursing home, Emergency department

## Abstract

**Background:**

OPTIC is a mixed method Partnership for Health System Improvement (http://www.cihr-irsc.gc.ca/e/34348.html) study focused on improving care for nursing home (NH) residents who are transferred to and from emergency departments (EDs) via emergency medical services (EMS). In the pilot study we tested feasibility of concurrently collecting individual resident data during transitions across settings using the Transition Tracking Tool (T3).

**Methods:**

The pilot study tracked 54 residents transferred from NHs to one of two EDs in two western Canadian provinces over a three month period. The T3 is an electronic data collection tool developed for this study to record data relevant to describing and determining success of transitions in care. It comprises 800+ data elements including resident characteristics, reasons and precipitating factors for transfer, advance directives, family involvement, healthcare services provided, disposition decisions, and dates/times and timing.

**Results:**

Residents were elderly (mean age = 87.1 years) and the majority were female (61.8%). Feasibility of collecting data from multiple sources across two research sites was established. We identified resources and requirements to access and retrieve specific data elements in various settings to manage data collection processes and allocate research staff resources. We present preliminary data from NH, EMS, and ED settings.

**Conclusions:**

While most research in this area has focused on a unidirectional process of patient progression from one care setting to another, this study established feasibility of collecting detailed data from beginning to end of a transition across multiple settings and in multiple directions.

## The Older Persons’ Transitions in Care (OPTIC) study: pilot test results

## Background

Despite the growing number of studies addressing problems in caring for older adults transferred between institutions at times of urgent need, progress in quality improvement during transitions has been hindered by challenges in measurement and attribution [1]. Current studies that assess outcomes of transitions of care have focused on patient readmission rates [2-4], adverse events [5,6] and the Care Transition Measure [7,8]. These outcomes do not address the complexity of transitions as they do not evaluate the entire transition process; rather, they evaluate the transition from one care setting to another. None of these studies evaluated whether a successful outcome was achieved in the transition process from the perspective of residents or multiple stakeholders.

In a recent report from the Canadian Institute for Health Information [9], 10% of all seniors’ (75+ years of age) admissions to acute care were nursing home (NH) residents, who overall had the longest waits for admission and highest lengths of stay and levels of readmission after discharge, making these admissions resource intensive. Much of the knowledge about use of emergency departments (EDs) by NH residents, however, arises from retrospective record reviews and a focus on effects of residents on the ED [10]. North American studies have reported a yearly transition incidence ranging from 23-60% from NH to ED [11-13]. A Canadian study that reported a 60% transition rate found of those 60%, 30% were admitted to hospital [12]. While the majority of NH residents sent to hospital for medical care return to their NHs, many experience multiple transfers among care settings and providers [14]. For example, Callahan et al. [15] found that ‘compound’ transitions occurred in 20% of transitions for dementia patients, each of which presented a new risk for communication errors, duplication of services, medical errors, and provision of care in conflict with the individual’s or family’s goals of care. Indeed, more generally transfers among the NH population are fraught with errors, inefficiency, suboptimal care and unmet care needs [16-19]. Therefore, studies are needed where a transition can be evaluated as a continuous process, corrected through system change, and includes perspectives of sending, interim and receiving providers, residents, and their families [17,20]. The development and use of well-designed tools to track processes, events and communications throughout a transition will provide data and advance understanding to make recommendations for change to improve outcomes and quality.

The OPTIC study is a three-year observational study funded by a Canadian Institutes of Health Research (CIHR) Partnerships for Health System Improvement (PHSI) grant (http://www.cihr-irsc.gc.ca/e/34348.html). This funding model requires participation and engagement by researchers and healthcare decision-makers from the genesis of ideas driving the project, to funding, and knowledge utilization to effect system change. This study examines the care that residents (aged 65 and older) receive when transferred from NHs via an emergency call to emergency medical services (EMS), to EDs and back. The objectives of the overall OPTIC study are to analyze all transfers between NHs and two EDs in two cities over a one-year period, to improve care, minimize complications, reduce stress on residents, families, staff and resources, improve management of resident transfers, and to develop a tool to measure transition success. In order to analyze transitions, the OPTIC team developed the Transition Tracking Tool (T3) [21], which facilitates detailed concurrent tracking of case-related data for transitions by individual NH residents beginning with the decision to transfer from the NH to the ED and ending with the resident’s return to the NH or death. The purpose of the OPTIC pilot study was to test feasibility of concurrently collecting individual resident data during transitions across these three settings using the T3. We had four process objectives (#1-4) and one outcome objective (#5) to assess the feasibility of:

1. Recruiting facilities/service providers in each of three study settings;

2. Enrolling participants;

3. Accessing and extracting data elements in each setting from patient care records;

4. Determining necessary revisions to the T3 tool, and

5. Describing the sample of transitions

## Methods

The OPTIC study protocol has previously been reported [21]. It includes the development of the T3 by OPTIC researchers and decision-makers, the OPTIC conceptual framework of the transition tracking process across three care settings (NH to ED via EMS and then return to NH via inter-facility transport services) and definitions of key terms in the T3. This transition process mapping from NH to ED was done to guide the study design, data collection, and analyses [21].

Briefly, the overall OPTIC study has three phases occurring over 42 months (November 2009 to April 2013). In Phase 1, qualitative data were collected from three stakeholder groups (residents and families, frontline healthcare providers, and managers/administrators) from three settings in each province (n = 71 participants) [22]. Our intent was to develop indicators of successful transitions [21]. In Phase 2, we undertook a one-year data collection phase using multiple tools including the T3 with a minimum target of 400 residents experiencing care transitions. This phase began with a three-month pilot of the T3 reported here. In Phase 3, we will complete analysis, interpretation, and knowledge translation of the study.

### Setting

The OPTIC pilot study was conducted in Kelowna, British Columbia (BC), and Edmonton, Alberta (AB). Kelowna’s 2011 population was 117,312, which represents a percentage increase of 9.6% since 2006 which is higher than the national average (5.9%) for this time period [23]. Kelowna’s population is among the oldest in the country with 19.1% aged 65+, compared to the relatively young population of Edmonton (11.7%) and Canada as a whole at 14.8% in 2011 [23]. Organization of healthcare services also differs between regions; 13 NHs served by a single ED in Kelowna, and 37 NHs served by seven EDs in Edmonton (see detailed description in [22]). These contrasts allowed for assessment of feasibility of collecting pilot data using the T3 in different healthcare settings and populations.

### Sample

Purposive, convenience samples were drawn from NH facilities.

#### Facility recruitment

In AB, all 37 NHs, one of the seven EDs, EMS and an inter-facility transfer service (IFTS) were approached to participate. Twenty five NHs agreed to participate in the OPTIC pilot, of which seven were voluntary, eight were public, and 10 were private. The AB ED, EMS and IFTS were publically owned and operated. In BC, all 13 NHs, ED, BC Ambulance Service (BCAS) and IFTS were approached to participate. Of the 12 participating NHs in BC, four were publically owned and operated, seven were private and one was voluntary. The BC IFTS was privately operated, while the ED and BCAS were publically owned and operated.

#### Resident enrolment

All residents aged 65 and older transferred via EMS from participating NHs to a participating ED were eligible for inclusion. We targeted the first 25 complete cases in each province or all cases completed between April to June, 2011 (whichever came first) to assess the feasibility of data collection tools and procedures.

### Ethics

A majority of Canadian NH residents (approximately 60% [9]) have some level of cognitive impairment, rendering many incapable of consenting to participate on their own behalf [24]. Consistent with Tri-Council policy, a waiver of consent was obtained to enroll all NH residents experiencing emergency transfers [21]. In BC, a full waiver of consent was obtained from the regional health authority for transferred residents in nine of the 12 participating NHs. For the remaining three NHs, while waiver of consent was granted to collect data at the hospital, operational approval required that residents (with a Cognitive Performance Scale score of 0–2) or their family members (if the resident had a Cognitive Performance Scale ≥3) [25] provide written consent prior to research staff accessing their NH care record. In AB, all participating NHs granted full waiver of consent. Ethics approval was obtained from the University of Alberta Health Research Ethics Board (HREB B: Pro00010666; Pro00017240) for AB and Interior Health Research Office and Research Ethics (UBCO BREB: 2010–017) as well as the University of British Columbia Okanagan Behavioural Research Ethics Board (UBCO BREB: H10-00127) for BC.

### Measure

The T3 is an electronic data collection tool created to obtain case-related data about residents to track processes [21], events and communications among healthcare providers [26] during their transition. The T3 is comprised of the following elements [21]:

*NH*: Demographic and medical, reasons for transfer, decision and timing of transfer, documentation that accompanied the resident during transition, and assistive technologies and devices (ATDs) (e.g., eye glasses, cane, hearing aids);

*EMS*: Canadian Triage and Acuity Scale (CTAS) scores [27] documentation received from NH, and prepared or received for ED, and timing information during transfer (notification and actual transfer times, arrival at ED);

*ED*: Timing information, from arrival at ED to assessment by nurse and doctor, consultation, diagnostic tests, chief complaint(s), reason(s) for admission, and overall length of ED stay (sub-divided into admission to inpatient bed and discharged from ED). We did not record all details during inpatient stays or for those transferred to a non-NH setting. However we did continue to track individuals once discharged from the inpatient unit.

*Disposition*: Resident’s location following transfer to ED (admission to inpatient care, return to original NH, transfer to another NH, or death);

*Discharge from ED/Inpatient IFTS/EMS*: Quality of communication between ED/Inpatient and IFTS, documentation sent during transfer, times of notification and actual transfer;

*Return to NH:* Medical follow-up information, ED/Inpatient data, documentation sent during transfer from ED/Inpatient via IFTS/EMS, update of resident’s medical list and patient care recommendations, and clinical assessment.

### Data collection procedure

For eligible residents, OPTIC staff collected transition information via on-site access to records (paper-based health records, electronic health records and patient care plans) at NHs and EDs using an electronic T3 version. This electronic version was programmed by Nooro Online Research (https://nooro.com) for desktop and wireless device (iPAD or laptop) application. Data were entered directly into wireless devices in care settings and automatically uploaded to a secure database. This significantly reduced workload and data entry error as paper copies were not used, eliminating the need for data re-entry into an electronic database. The electronic application was tested extensively prior to field use. Data collection issues and procedure standardization were managed at regular team meetings among research staff and investigators from both provinces.

### Analysis

Data were analyzed using IBM SPSS Statistics 20 (SPSS Inc., Chicago, IL). Percentages are reported for categorical data; medians, means and standard deviations for continuous data. Data are reported for both provinces combined. While we plan to compare differences in resident characteristics, transitions and outcomes across both provinces, the pilot sample size was too small to make meaningful comparisons. Variation in the number of missing cases by item resulted in variation in denominators. The relevant denominator is therefore reported for each data point. Missing data patterns were analysed by preparing a missing data report (% missing) for first-order questions (no skip patterns or secondary data) to identify data elements that required further work and cases to be excluded if more than 50% of data was not retrieved across all settings.

## Results

### Resident sample description

Of 114 cases identified during the 3 month pilot period (AB = 85, BC = 29), 54 transitions (AB, N = 28; BC, N = 26) had data from each setting (NH, EMS, ED) and were included in the pilot study. In Alberta, 57 cases were identified in the ED but either did not meet the inclusion criteria (from assisted living facilities; facilities for which we had not yet received operational approval); or, we could not retrieve data from each setting in the transition. Resident transitions included were from 10 AB NHs (range 1-6 cases per NH) and 8 BC NHs (1–8).

A majority of the 54 residents were female (61.8%) and mean age was 87.1 years (SD = 6.9) ranging from 71 to 100 years. Residents frequently had multiple health challenges ranging from 0 to 7 per resident, with 47.2% having four or more. The most commonly recorded impairments were vision (59.6%), cognition (55.8%), activities of daily living (50.0%) and mobility (48.1%). Nearly two-thirds of residents had legal/proxy substitute family decision-makers (64.8%). Almost all transferred residents (>95%) had next of kin identified in the resident’s NH chart. Demographic data were complete and accessible.

### Process objective 1: recruiting facilities/service providers in each of three study settings

A total of 25 NHs in AB (73%) and 12 NHs in BC (92%) agreed to participate in the pilot study. Several AB NHs were still in the process of providing operational approval when the pilot study began. Both AB and BC EDs agreed to participate and provide data collection assistance. EMS in AB and IFTS in both provinces agreed to participate, while BCAS declined.

### Process objective 2: enrolling participants

Strategies to enroll residents differed by province. In BC, research staff provided NH managers/delegates with a notification script and designated phone number to call to leave a message on a password protected voicemail when a resident (aged 65 or older) was sent via ambulance for an emergency transfer to the ED. In AB, the initial notification process involved EMS notifying research staff about a transfer from participating NHs to ED. EMS was chosen for two reasons - they were going to the NH and knew which ED the resident went to. In both provinces most data elements were collected from resident health records. The main sources of data were NH charts, EMS patient care record (PCR), ED chart and inpatient records as applicable. Some data were accessed electronically, for example, the Emergency Department Information System (EDIS) in AB.

### Process objective 3: accessing and extracting data elements in each setting from patient care records

#### Missing data

Each study site and data element had varying degrees of missing data. The most frequently missing data elements were documentation of Assistive Technologies and Devices (ATD - e.g., eye glasses, canes, dentures) accompanying the resident from one setting to the next. Whether these were sent with residents was rarely recorded in any OPTIC study site. Information related to the decision to transfer including trigger events and involvement in conversations to transfer was more complete (e.g., 7.4% missing for trigger events). Information on times between events during transitions was not complete (e.g., 24.1% missing for time of arrival at ED to being seen by a nurse). ED data were generally most complete of all study sites (e.g., no missing cases for consultations, diagnostics tests; 14.8% missing for final diagnosis). Documentation data were least accessible in originating NHs (NH1) (26.0% missing) and on return to NH post-transition (NH2) (33.3% missing). EMS data from NH1 to ED (EMS1) (13.0% missing) were most often accessible as were ED data (5.6% missing). Data were missing most often for the resident’s return trip from ED to NH2 via IFTS (46.3%). This is likely due to a problem with data elements missed in programming the T3 electronic data collection form. Feasibility of data collection was thus variable and subject to alterations in data collection protocol.

#### Time to complete the T3

Assuming ideal circumstances, where data were immediately available to research staff, T3 completion time would have been 2–3 hours. However, actual completion time of an individual transition from start to finish routinely took much longer and varied widely between cases. There were several reasons for this. First, complexity of resident transitions led to significant differences in time for completion depending on the resident’s medical needs. For example, it took longer to track transfers when a resident was admitted to an inpatient unit rather than discharged back to NH. Second, OPTIC staff tracked multiple transfers at once, complicated in AB because not all NH operational approvals had yet been secured. This resulted in retaining cases enrolled with ED operational approval until NH operational approval to collect data was received. Third, a number of factors made it difficult to access residents’ charts, e.g. if research staff were interrupted or had difficulty accessing hospital medical records or NHs charts, resulting in multiple trips to hospitals or NHs to collect all data. Fourth, logistics were an important consideration in AB due to the large number of NHs providing operational approval and their geographic spread across the city. It was important to coordinate data collection for transfers from one NH or NHs located in the same vicinity to optimize efficiency. Thus, data collection for some transfers was delayed until it was possible to attend the NH.

### Process objective 4: determining necessary revisions to the T3

The pilot study allowed the OPTIC team to identify and rectify “glitches” in the T3 such as missing “other”, “not applicable” and/or “not recorded” options, and incorrect skip pattern questions. Regarding specificity of time elements, we provided the opportunity for data collectors to indicate whether an event occurred during the day (0700–1500), evening (1500–2300), or night (2300–0700) shift when exact times were not recorded. Additional field note sections were added to allow research staff to provide contextual notes where appropriate, and a data dictionary was developed to define each term. No data elements were added or deleted. Due to the comprehensive nature of data collection and frequent travel to ED and NH sites, additional research assistants were contracted in each province. Two additional iPads were acquired and configured to allow a secure online server to store both complete and incomplete files. All transition tracking data were stored on secure servers with security configured such that each case could be accessed by one OPTIC staff member at a time. For example, one data collector could download a case while in the ED to enter data on the resident’s admission. Once that case was uploaded to the server, another data collector in the NH could download the updated case on the resident’s return and add additional information.

The initial process of EMS identifying residents being transferred to the ED in Alberta and then notifying research staff did not work. The process was revised to have research staff access the Emergency Department Information System (EDIS) to identify new eligible transfers. In BC while the initial identification process worked, significant staff support was required to remind NH staff about the study, and simultaneously inquire about transfers that had occurred.

Due to the large number of participating facilities and staff at each site, research staff attempted to locate and establish a working relationship with a contact person(s) at each site. When these relationships were developed, data collection was more efficient and complete as contact persons provided OPTIC research staff with access to charts and other required information. Each study site used a different charting system; even NH health records varied by facility. During the pilot, data collectors learned where to look in residents’ charts for specific data elements in the T3.

### Outcomes objective 5: describing the sample of transitions

#### The resident at the NH (NH1): the beginning of a transition

A trigger event – either a change in resident’s condition and/or an acute event – caused one or more individuals to be consulted about possible transfer, with one individual making the final decision to initiate transfer (see Table 1). Falls causing injury were the most common trigger event (30.9% of all trigger events), followed by a change in physical condition (14.7%), and nausea/vomiting/diarrhea (11.8%). Between one and three trigger events were recorded for each resident. Trigger event data were available and accessible for 50 of the 54 residents.

**Table 1:** Events and decisions leading to transitions

**Trigger event(s)**	**N (%)**
Falls	21 (30.9)
Fractures and other fall related injuries	4 (5.9)
Hip/pelvis/leg pain	1 (1.5)
Change in physical condition	10 (14.7)
Nausea/vomiting/diarrhea	8 (11.8)
Change in mental status	5 (7.4)
Shortness of breath	3 (4.4)
Family/friend caregiver request	3 (4.4)
Chest Pain	3 (4.4)
Urinary symptoms	3 (4.4)
Change in behaviour	2 (2.9)
Respiratory symptoms	2 (2.9)
Other	4 (5.9)
# of trigger events	68
Valid cases/missing	50/4
**Who was involved in decision to transfer?** ** (Check all that apply)**	
Registered Nurse	32 (38.1)
Physician of record	20 (23.8)
LPN	16 (19.0)
Family/friend caregiver	10 (11.9)
Resident	2 (2.4)
Nurse practitioner	2 (2.4)
Physiotherapist	1 (1.2)
Healthcare Aide (HCA)	1 (1.2)
# involved	84
Valid cases/missing	50/4
**Who made the final decision to transfer?**	
Physician of record for resident	16 (33.3)
Registered Nurse	12 (25.0)
Family/Friend	9 (18.8)
Licenced Practical Nurse	7 (14.6)
Resident	2 (4.2)
Nurse practitioner	2 (4.2)
Valid cases/missing	48/6

NH staff most frequently involved in discussions concerning decision to transfer were Registered Nurses (RN; 38.1%), the physician of record (23.8%), Licensed Practical Nurses (LPN; 19.0%) family/friend caregiver (11.9%), and residents (2.4%). The resident’s physician most often made the final decision to transfer (33.3%), followed by RNs (25.0%), family/friend caregiver (18.8%), LPNs (14.6%), and residents themselves (4.2%).

#### Emergency medical services: from NH to ED (EMS1)

**Figure 1:**
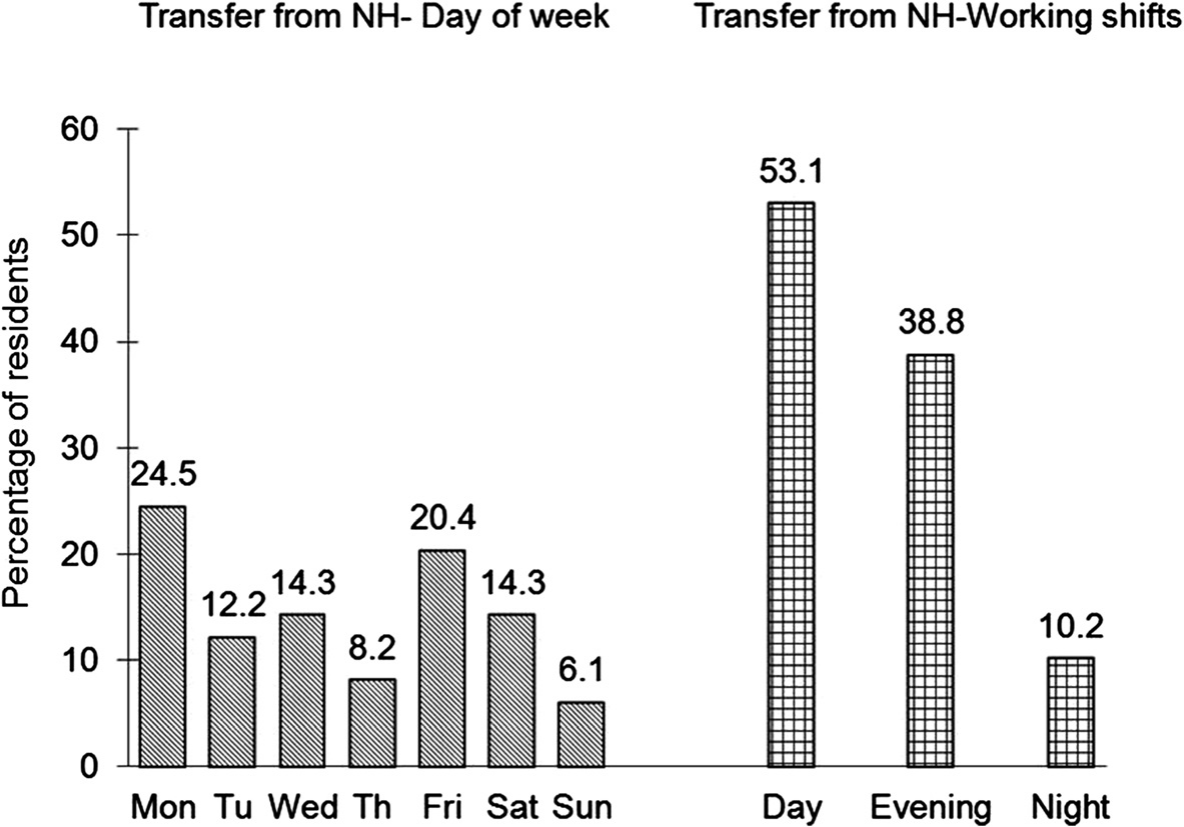
Transfer day of the week and shift (day, evening and night).

Transitions occurred each day of the week, with almost half on Mondays (24.5%) and Fridays (20.4%). Transfers took place most often on day shift (0700–1500, 53.1%), evening shift (between 1500–2300, 38.8%) and night shift (2300–0700, 10.2%). Figure 1 shows the distribution of ED transfers by day of week and by shift.

#### Emergency department

Select timing variables following arrival at ED are presented in Table 2. Median time between arrival and being seen by a nurse was 70 minutes (N = 41), and to be examined by an ED physician was 73 minutes from arrival at ED (N = 28). Median time for the decision on disposition (arrival at ED to decision to admit to hospital or discharge to original NH (N = 28)) was 394 minutes. Median length of stay in the ED for residents not admitted as inpatients was 468 minutes (N = 18), compared to 529 minutes for residents admitted to inpatient units (N = 17). For the latter, median time between arrival at ED and return to NH was 5 days, 6.5 hours (N = 15).

**Table 2:** Time for resident to be seen from arrival time at the ED (hours: minutes)

**Time from arrival at the ED to**	**N**	**MDN**	**Mean (SD)**
Being seen by a nurse	41	1:10	3:54 (10:53)
Being seen by a physician (history and examination details recorded)	28	1:13	1:40 (1:08)
Decision to admit or return to NH	28	6:34	11:00 (20:31)
Actual admission to the inpatient unit	17	8:49	14:08 (10:36)
Actual transfer back to NH (admitted to inpatient unit) (total time spent in ED and hospital)	15	126:29	196:01 (170:25)
Actual transfer back to NH (not admitted) (total time spent in ED)	18	7:38	10:54 (10:53)

Prior to ED admission, all residents were assessed using CTAS to determine urgency for treatment. The scale score ranges from 1 to 5 (5 = lowest priority for immediate treatment, 1 = life-saving resuscitation is needed). In BC, CTAS scores were assigned by RNs; in AB, CTAS scores were assigned by EMS personnel or RNs (84.0%, 16%, respectively). Most residents (74.0%) were scored 3, which indicated a non-life threatening condition requiring immediate action.

Upon admission to ED, consultations, diagnostic tests and medical procedures (Table 3) were completed. Fewer than half of residents (40.7%, n = 22) received specialty consultative services and 10 (18.5%) received consultations from two or three services. Internal Medicine was the most common (25.9%), followed by orthopaedics (14.8%), gastroenterology and gerontology (11.1% each). All residents had one or more diagnostic tests performed (mean = 2.4 tests). The three most common diagnostic tests conducted were lab work (27.1% of the total tests performed), X-rays (25.6%), and urinalysis (20.9%). Almost half of transferred residents underwent medical procedures (46.3%).

**Table 3:** Assessments, care and services while in the ED (n = 54)

**CTAS score**	**N (%)**
I	0 (0.0)
II	7 (14.0)
III	37 (74%)
IV	5 (10.0)
V	1 (2.0)
Valid cases/missing	50/4
**Service(s) provided**	
Consultation(s)	22 (40.7)
Diagnostic test(s)	54 (100.0)
Medical procedure(s)	25 (46.3)
Valid cases (for each service)	54
**Consultation(s)**	
Internal medicine	7 (25.9)
Orthopaedics	4 (14.4)
Gastroenterology	3 (11.1)
Gerontology	3 (11.1)
Other	10 (37.0)
Total consultations	27
Valid cases (for each consultation type) /Missing (no consultation)	22/32
**Diagnostic test(s)**	
Lab work	35 (27.8)
X-rays	33 (26.2)
Urine	27 (21.4)
Electrocardiogram	13 (10.3)
CT scan	9 (7.1)
Ultrasound	3 (2.4)
Radiometer	2 (1.6)
Other	7 (5.6)
Total consultations /Valid cases (for each test type)	126/54
**ED Final Diagnosis**	
Fractures (hip/pelvis, limb)	12 (26.1)
Falls related injuries	8 (17.4)
Respiratory (respiratory failure, pneumonia, COPD)	5 (10.9)
Urinary-related illness	4 (8.7)
Altered mental status (dementia, confusion)	4 (8.7)
Gastro-intestinal (GI bleed, bowel obstruction)	3 (6.5)
CVA (stroke)	3 (6.5)
Cardiac (cardiac arrest, chest pain, CHF)	2 (4.3)
Abnormal blood work	2 (4.3)
Other	3 (6.5)
Valid cases/missing	46/8

Final ED diagnosis was recorded for 85.2% residents. Fractures were most common (26.1%), followed by falls related injuries (17.4%) and respiratory conditions (10.9%). Delirium was infrequently recorded during transitions. In three cases, the chief complaint on arrival to ED was recorded as follows: bizarre behaviour (n = 1) with a final diagnosis of query delirium, and confusion (n = 2) with final diagnoses of dementia and iron toxicity respectively. We collected data on disposition decision and actual disposition of resident. Fewer than half (43.4%) of residents were admitted as inpatients, while 54.7% were discharged back to their originating NH. One person was sent to a different NH. Three residents (5.7%) died while in inpatient care.

### Transport back to NH

Data were available for 44 residents. IFTS was the most common mode of transport back to a NH (86.4%), with the remainder by EMS, EMS Patient Transfer or family/friend caregiver.

### Tracking of Assistive Technologies and Devices (ATD)

One component of the T3 was designed to capture whether or not nine specific ATD were recorded as accompanying the resident through each transition stage, including: glasses, dentures, hearing aids, medications, healthcare card, cane/walker clothing, slippers, and an “other” category to capture items not on this list. Overall, ATDs were rarely recorded throughout the transition process.

### Tracking accompanying documentation

Appropriate transfer of critical documentation was tracked across transition settings; however, the number of cases where data were available differed by study site. Frequencies of resident documentation being sent across two or more stages of a transition are presented in Table 4, which in turn can be used to identify sources of information gaps across care settings. Medication lists were recorded as sent most often in the transfer to ED rather than on return to NH. Records of allergies, do not resuscitate (DNR) orders, patient care plans and advance directives were passed from the originating NH through all settings back to the NH.

**Table 4:** Documentation accompanying resident at each stage

	**Stage of transition**^**a**^				
	**NH1**	**EMS1**	**ED**	**EMS2**	**NH2**
**Documentation**	**N (%)**	**N (%)**	**N (%)**	**N (%)**	**N (%)**
Medication list	33 (82.5)	47 (100.0)	48 (94.1)	^c^	18 (50.0)
Patient summary and transfer Information	29 (72.5)	42 (89.4)	41 (80.4)	^c^	6 (16.7)
Record of allergy	27 (67.5)	38 (80.9)	39 (76.5)	^c^	8 (22.2)
DNR Order	23 (57.5)	31 (66.0)	34 (66.7)	^c^	6 (16.7)
Advance directive	17 (42.5)	19 (40.4)	17 (33.3)	^c^	2 (5.5)
Patient Care Plan	11 (27.5)	11 (23.4)	17 (33.3)	^c^	5 (13.9)
Resident Data	8 (20.0)	18 (38.3)	16 (31.4)	^c^	1 (2.8)
Physician Orders & Notes	3 (7.5)	7 (14.9)	5 (9.8)	^c^	1 (2.8)
Resident Clinical Data	2 (5.0)	4 (8.5)	8 (15.7)	^c^	3 (8.3)
List of Diagnoses	3 (7.5)	5 (10.6)	6 (11.8)	^c^	
PCR form	^b^	^b^	44 (86.3)	^c^	3 (8.3)
ED summary	^b^	^b^	^b^	19 (65.5)	14 (38.9)
Inpatient summary	^b^	^b^	^b^	8 (27.6)	8 (22.2)
Transfer record	^b^	^b^	^b^	7 (24.1)	8 (22.2)
Lab results/orders	^b^	^b^	^b^	^c^	9 (25.0)
ED Nurses’ Notes	^b^	^b^	^b^	^c^	8 (22.2)
Patient follow up	^b^	^b^	^b^	^c^	3 (8.3)
Inpatient forms	^b^	^b^	^b^	^c^	3 (8.3)
Consultations	^b^	^b^	^b^	^c^	3 (8.3)
Inpatient Nurses’ Notes	^b^	^b^	^b^	^c^	3 (8.3)
Follow up appointments	^b^	^b^	^b^	^c^	2 (5.5)
OR documentation	^b^	^b^	^b^	^c^	3 (8.3)
Other	2 (5.0)	1 (2.1)	4 (7.8)	^c^	0 (0.0)
Valid cases (for each type of documentation)	40	47	51	29	36
Missing	14	7	3	22^d^	15^d^

^a^NH1 = originating nursing home; EMS1 = emergency medical services to ED; ED = emergency department; EMS2 = transportation service from ED; NH2 = return to nursing home.

^b^not applicable.

^c^not recorded in the T3.

^d^3 residents died in inpatient care.

The ED summary, inpatient summary, transfer record, lab results/orders, patient follow-up and others were not commonly recorded or found in the resident’s NH chart upon return. All documentation types were missing most often for the return trip via IFTS/EMS to the NH (NH2).

## Discussion

Coleman defined ‘transitional care’ as “a set of actions designed to ensure the coordination and continuity of healthcare as patients transfer between different locations or levels of care in the same location” [28]. While most research in this area has focused on the progression of patients from one care setting to another as a unidirectional process, the reality is that transitions occur across multiple settings and in multiple directions [5,10,29]. Despite the challenges identified during the course of this pilot study, concurrent case tracking during transitions was feasible.

Few NH to ED transition studies have been conducted in Canada (e.g. see [11,16,30]) and much of the literature is limited to one part of the system – the ED. Few focus on pre- and post-hospital transfer services. Each care setting tends to behave in a “silo” manner, resulting in difficulty with relationship formation and limits to access to vital patient information which can compromise cross-site care coordination [31]. This pilot study is unique in that it followed residents throughout their transition experiences across all care settings and captured in-depth data about the transition process not elsewhere available. An advantage of this approach is the opportunity to identify gaps in care as the resident moves through the different organizations that comprise the system. Previous research has indicated that a significant information gap often exists between NHs and the ED [32-34]. The T3 was designed to record the flow of all relevant documentation during the course of a transfer, allowing for the identification of the setting within which these gaps occurred. With one exception (discussed below) the T3 was successful in tracking the flow of documentation through each setting.

Consistent with the literature, the T3 pilot study found that the most common information gap in documentation sent between facilities were: assistive technologies and devices [32], laboratory test results [34] and cognitive function documentation [32-34]. These three studies [32-34] identified information gaps at single settings in the transition, whereas the T3 is able to identify information gaps that occur at each setting across the entire transition. The pilot study clearly illustrates the inconsistency of the content sent across settings and has the potential to help identify essential elements required across the entire transition as well as information deemed essential for each setting. To date, no known studies have identified essential information across the entire transition [32,34].

In both provinces, however, effective tracking of residents across settings necessitated site-specific strategies and strong relationships between research staff and facilities staff. The Partnerships in Health System Improvement model of conducting research (http://www.cihr-irsc.gc.ca/e/34348.html) requires close cooperation between researchers and decision-makers from the genesis of the idea through to meaningful knowledge translation of the results. Relationship building and maintenance are critical to successful data collection from planning to implementation [35]. Consistent with recommendations from the research literature (e.g. see [36]), regular face-to-face consultation sessions, meetings and other forms of communication formed the basis of these relationships. It is difficult to imagine the successful trialing of the T3 without such in-depth involvement and combined sense of purpose by both researchers and decision-makers.

The T3 tracked assistive technology and devices; however, tracking of ATDs was difficult due to inconsistent documentation in the residents’ care records at all points of the transfer. In a study by Hammel et al. stakeholders including patients and providers identified ATD use as decreasing demands on others for time, assistance, energy, mitigating safety risk and conducting activities of daily living and that this benefit far exceeded just functional independence [37]. Poor tracking and provision of ATDs during transitions has been a source of ED patient dissatisfaction and has accounted for up to a third of patient complaints [38]. In extreme cases, the loss of dentures (one ATD) in a NH has led to a documented adverse drug event [39]. Although this pilot study was not measuring cost, both the prohibitive cost of replacing these items for some and the difficulty or impossibility of replacing them in a population with dementia require further investigation. There are also implications for quality of life in the event that ATDs are not available to residents during their transition and beyond.

Any conclusions based upon outcome data in this pilot study (objective 5) are limited by the small sample size. Due to the dynamic nature of the process, some data could only be gathered a period of time after discharge from hospital. It is possible that not all information (e.g., communication, documentation, etc.) transferred with the resident was kept in the resident’s care record or was recorded in the chart at the time the research staff accessed residents’ records.

## Conclusions

Older adults transferred between NHs and EDs represent a group of patients at risk for errors and adverse medical outcomes. While collecting data on transitions from a NH to the ED and back is complex, it can be achieved. Overall, the T3 provided valuable and detailed information about transitions in care for elderly patients transferred from NH to EDs in two Canadian provinces. Further research will expand the sample size, provide detailed documentation of the transition issues facing elderly NH patients and provide recommendations for reducing gaps in care for this vulnerable population.

## Competing interests

The authors declare that they have no competing interests.

## Authors’ contributions

RCR participated in the design of the study, was provincial lead for the BC component of the study and was lead author on the writing of the manuscript. GEC was lead on the T3 phase of the OPTIC project, was EMS lead and participated in the design of the study and editing of the manuscript. SLC was second writer for the manuscript, prepared figures, provided editing and was responsible for preparation of the manuscript for publication submission. SA carried out data collection. SA and LJB carried out data collection. CAE participated in the design of the study, original conception of study, worked with GGC, PGN and RCR for funding of the study and editing of the manuscript. BHR provided access to ED for data collection as the AB ED lead for the project, participated in design of the study and editing of the manuscript. AW participated in design of the study. PGN participated in the design of the study, original conception of the study and worked with GGC, CAE and RCR to secure funding for the study. ME provided access to ED data collection as the BC ED lead for the project. ME also reviewed an early version of the manuscript. GGC is the nominated principal investigator for the OPTIC research program, provided leadership and coordination of the AB group, led its design and proposal development, and helped to draft the manuscript. All authors read and approved the final manuscript.

## Pre-publication history

The pre-publication history for this paper can be accessed here:

http://www.biomedcentral.com/1472-6963/13/515/prepub

## Principal investigators

Greta G. Cummings, Nominated Principal Applicant, University of Alberta

Carole A. Estabrooks, Principal Applicant, University of Alberta

Peter G. Norton, Principal Applicant, University of Calgary

R. Colin Reid, Principal Applicant, University of British Columbia Okanagan

## Co-investigators

Joan Bottorff, Co-Applicant, University of British Columbia Okanagan

Garnet E. Cummings, Co-Applicant, University of Alberta

Norah Keating, Co-Applicant, University of Alberta

Jacques S. Lee, Co-Applicant,

Sunnybrook Health Sciences Centre

Meredith Lilly, Co-Applicant, University of British Columbia

Candace Nykiforuk, Co-Applicant, University of Alberta

Belinda Parke, Co-Applicant, University of Alberta

Carole Robinson, Co-Applicant, University of British Columbia Okanagan

Brian H. Rowe, Co-Applicant, University of Alberta

Adrian Wagg, Co-Applicant, University College Hospital

## Principal decision-makers

Joanne Konnert, Chief Operating Officer, Interior Health

Glenda Coleman-Miller, Vice President, University of Alberta Hospital

## Decision-makers

Carol Anderson, Executive Director, Edmonton Zone, Alberta Health Services

Cindy Crane, Manager, Interior Health, Kelowna General Hospital

Michael Ertel, Chief and Medical Director, Interior Health Kelowna General Hospital

Karen Latoszek, Senior Manager, Alberta Health Services

Tracy Buffam, Client Services Manager, Good Samaritan Southgate Care Center

Corinne Schalm, Director, Access & Innovation, Continuing Care, Alberta Health

Cindy Regier, Director Residential Services, South Okanagan

Sunil Sookram, Medical Director EMS, Alberta Health Services

## Additional members

Faye Burch, Director Residential Services, Cottonwoods Care Centre

Sarah L. Cooper, Graduate Student, Research Assistant
